# Effect of atorvastatin on skeletal muscles of patients with knee osteoarthritis: *Post-hoc* analysis of a randomised controlled trial

**DOI:** 10.3389/fmed.2022.939800

**Published:** 2022-08-25

**Authors:** Yuan Z. Lim, Flavia M. Cicuttini, Anita E. Wluka, Graeme Jones, Catherine L. Hill, Andrew B. Forbes, Andrew Tonkin, Sofia Berezovskaya, Lynn Tan, Changhai Ding, Yuanyuan Wang

**Affiliations:** ^1^School of Public Health and Preventive Medicine, Monash University, Melbourne, VIC, Australia; ^2^Menzies Institute for Medical Research, University of Tasmania, Hobart, TAS, Australia; ^3^The Queen Elizabeth Hospital, University of Adelaide, Woodville, SA, Australia; ^4^Department of Medicine, University of Adelaide, Adelaide, SA, Australia; ^5^Alfred Hospital, Melbourne, VIC, Australia; ^6^Clinical Research Centre, Zhujiang Hospital, Southern Medical University, Guangzhou, China

**Keywords:** statins, osteoarthritis, knee, muscles, myalgia

## Abstract

**Objective:**

Populations with knee osteoarthritis (KOA) are at increased risk of cardiovascular disease, due to higher prevalence of risk factors including dyslipidaemia, where statins are commonly prescribed. However, the effect of statins on muscles and symptoms in this population is unknown. Thus, this study examined the effect of atorvastatin on muscle properties in patients with symptomatic KOA.

**Design:**

*Post-hoc* analysis of a 2-year multicentre randomised, double-blind, placebo-controlled trial.

**Setting:**

Australian community.

**Participants:**

Participants aged 40–70 years (mean age 55.7 years, 55.6% female) with KOA who met the American College of Rheumatology clinical criteria received atorvastatin 40 mg daily (*n* = 151) or placebo (*n* = 153).

**Main outcome measures:**

Levels of creatinine kinase (CK), aspartate transaminase (AST), and alanine transaminase (ALT) at 1, 6, 12, and 24 months; muscle strength (by dynamometry) at 12 and 24 months; vastus medialis cross-sectional area (CSA) on magnetic resonance imaging at 24 months; and self-reported myalgia.

**Results:**

There were no significant between-group differences in CK and AST at all timespoints. The atorvastatin group had higher ALT than placebo group at 1 (median 26 vs. 21, *p* = 0.004) and 6 (25 vs. 22, *p* = 0.007) months without significant between-group differences at 12 and 24 months. Muscle strength increased in both groups at 24 months without between-group differences [mean 8.2 (95% CI 3.5, 12.9) vs. 5.9 (1.3, 10.4), *p* = 0.49]. Change in vastus medialis CSA at 24 months favoured the atorvastatin group [0.11 (−0.10, 0.31) vs. −0.23 (−0.43, −0.03), *p* = 0.02] but of uncertain clinical significance. There was a trend for more myalgia in the atorvastatin group (8/151 vs. 2/153, *p* = 0.06) over 2 years, mostly occurring within 6 months (7/151 vs. 1/153, *p* = 0.04).

**Conclusions:**

In those with symptomatic KOA, despite a trend for more myalgia, there was no clear evidence of an adverse effect of atorvastatin on muscles, including those most relevant to knee joint health.

## Introduction

Osteoarthritis (OA) is a common cause of pain and disability. However, generally overlooked is the fact that people with OA die of cardiovascular disease (CVD) at approximately twice the rate of the general population (1, 2). This relates to the increased prevalence of CVD risk factors among those with OA, including dyslipidaemia (3).

Statins, one of the most widely prescribed drug classes worldwide, have the well documented benefit of reducing coronary heart disease events and stroke, by lowering the levels of low-density lipoprotein cholesterol (4). Statins have been the cornerstone of pharmacotherapy for the management of dyslipidaemia virtually since their development (5). They are generally safe and well tolerated (6). Nevertheless, statin-associated muscle symptoms, present most commonly as myalgia and rarely as myopathy, myositis or rhabdomyolysis, have been cited as the most common reason for statin discontinuation (7, 8). In a survey of 10138 statin users, while most patients (62%) discontinued statin therapy due to side effects, nearly 1/3 stopped their statin therapy due to muscle related side effects without consulting their clinicians (7), possibly due to distortion of the risk-benefit ratio and hence unduly concerns about potential harms of statins from non-clinician sources (9). The prevalence of statin-induced muscle symptoms varies, depending on how it is defined and assessed. There is a huge discrepancy in the incidence of myalgia, ranging from 1 to 5% in clinical trials to 11–29% in observational cohort studies (10). The National Lipid Association Task Force on Statin Safety 2014 update highlighted the limitation of using current evidence of safety from randomised controlled trials because such populations are typically very restricted in their study entry characteristics, excluding patients with multiple comorbidities, previous statin intolerance, and people with active musculoskeletal conditions (10). In addition, varying definitions for statin-associated muscle symptoms have been used (8, 10).

Muscles play an important role in the prevention and management of knee OA (11). Muscle weakness has been associated with the development and progression of knee OA. In patients without radiographic knee OA, weak knee extensor strength has been associated with increased risk of developing symptomatic knee OA (12) while in patients with established radiographic and symptomatic knee OA, weak knee extensor is associated with increased risk of symptomatic and functional deterioration (13). There is evidence that statin use may exacerbate the age-related decline in muscle performance and increase the risk of falls despite no reduction in muscle mass in community-dwelling older adults (14). Hence, it is possible that statin-associated muscle symptoms may worsen the tolerability of statin in patients with OA. Conversely, individuals with OA are at twice the risk of CVD mortality (1) and therefore at greater need for statin. As those with symptomatic OA are excluded from clinical trial of statins, the effect of statin on skeletal muscles in populations with symptomatic OA is unknown. Thus, the aim of this study was to examine the effect of atorvastatin on skeletal muscle properties (biochemistry, strength, size, and myalgia) in a *post-hoc* analysis of a randomised controlled trial examining the effect of atorvastatin on progression of knee OA (15).

## Materials and methods

### Study design and participants

The Osteoarthritis of the Knee Statin (OAKS) study was a 2-year multicentre randomised, double-blind, placebo-controlled trial evaluating whether atorvastatin had a disease-modifying effect in patients with symptomatic knee OA (15, 16). In brief, eligible participants aged 40–70 years with symptomatic knee OA for ≥6 months with a pain score of >20 mm on a 100 mm visual analog scale, and who met the American College of Rheumatology clinical criteria for knee OA (17) were enrolled. Exclusion criteria were severe radiographic knee OA [grade 3 joint space narrowing according to Altman’s atlas (18)]; severe knee pain (on standing >80 mm on 100 mm visual analog scale); inflammatory arthritis; accepted indications for statin therapy, including familial hypercholesterolaemia, known atherosclerotic cardiovascular disease, and diabetes mellitus; current use of lipid-lowering therapy, or previous adverse reaction to statins; absolute cardiovascular risk estimated using the Framingham Risk Equation of >15% within the next 5 years; fasting total cholesterol level >7.5 mmol/L; clinically significant renal disease or abnormal liver function. Ethics approval was obtained from Alfred Hospital Ethics Committee, Monash University Human Research Ethics Committee, Tasmania Health and Medical Human Research Ethics Committee, and The Queen Elizabeth Hospital Human Research Ethics Committee. All participants provided written informed consent. The trial was registered with Australian New Zealand Clinical Trials Registry (ACTRN12613000190707).

### Study protocol

Participants were randomly assigned in 1:1 ratio to receive either 40 mg atorvastatin once daily or inactive matching placebo once daily. Details concerning randomisation and masking have been reported previously (15, 16). All participants were provided usual care by their treating health practitioners. At screening, participants completed questionnaires, had a knee X-ray, and underwent biochemical testing including liver function tests, creatine kinase (CK) and renal function tests, to ensure inclusion criteria were met. Height and weight were measured at baseline. Subsequent study visits were scheduled at 6, 12, and 24 months. Adverse events were monitored throughout the trial. Participants were requested to report any adverse event at each study visit and by phone calls outside the scheduled study visits. Serious adverse events were determined by a rheumatologist who was blinded to treatment allocation. Details of the adverse event and its relationship with the intervention were recorded and reported to the Ethics Committees. The primary outcome of the OAKS study was the annual percentage change in tibial cartilage volume, measured by magnetic resonance imaging (MRI) (15).

### Muscle biochemistry

Biochemical testing including CK and liver function tests [alanine transaminase (ALT), aspartate transaminase (AST)] were performed at screening, 4 weeks, 6, 12, and 24 months for safety monitoring, according to the manufacturer’s instruction in accredited commercial laboratories. All abnormal biochemistry results were reviewed by a rheumatologist to determine the clinical significance, relevance, and appropriate management.

### Muscle strength

Muscle strength was measured by dynamometry to the nearest kilogramme in both legs simultaneously at baseline, 12 and 24 months (14). The muscles measured in this technique are mainly quadriceps and hip flexors. The technique has been previously described (14). Three readings were recorded, and the highest score was used. The devices were calibrated by suspending known weights at regular intervals. Repeatability estimates (Cronbach’s) were 0.91 (19).

### Muscle size

Magnetic resonance imaging of the study knee was performed at baseline and 24 months using 1.5T or 3T whole-body MRI units with a commercial transmit-receive knee coil. Details of MRI units, sequences and parameters have been published (16). Cross-sectional area (CSA) of vastus medialis, a central muscle responsible for knee joint stability and function (11, 13), was measured on axial MRI images (11, 20). The CSA of vastus medialis was measured specifically at the MRI slice 37.5 mm superior to the quadriceps tendon insertion at the proximal pole of the patella, orthogonal to the long axis of the leg. The muscle boundary was manually traced using the OsiriX Software. Baseline and follow-up MRIs were read paired by one trained observer, blinded to group allocation, participant characteristics, and time sequence of MRI. The intraobserver reproducibility (intraclass correlation coefficient, ICC) of the measurement was 0.95.

### Muscle symptoms

Participants who self-reported myalgia through adverse events monitoring were assessed by a rheumatologist and managed on a case-by-case basis.

### Statistical analysis

Participant characteristics at baseline were tabulated and compared between the atorvastatin and placebo groups using independent samples *t*-test, Mann-Whitney *U*-test, or chi square test, as appropriate. Per protocol analyses of all the outcome measures were performed according to the participants’ randomised treatment group restricted to those with available outcome measures at different timespoints. Independent samples *t*-test and Mann-Whitney *U*-test was used to compare muscle measures between the two groups at each time point, when appropriate. Muscle biochemistry biomarkers, strength, and CSA were compared between the atorvastatin and placebo groups by using a repeated measures mixed-effects linear regression model with terms of treatment, time, sex and corresponding baseline values as covariates. The correlations within the repeated measures were addressed by using the participants’ randomisation identification as a random effect. The effect of treatment at baseline and week 4, months 6, 12, and 24 was evaluated by adding an intervention-by-time interaction to the regression models. The linear mixed-effects model incorporates all the study participants and assumes that data are missing at random. Chi square test or Fisher’s exact test was used to compare the incidence of myalgia between the two groups. With 248 participants completing the 2-year follow-up, the study had 80% power to detect a difference of 5 kg in muscle strength change, a difference of 0.5 cm^2^ in muscle CSA change, and a difference of 6 units in muscle biochemistry biomarker change between the atorvastatin and placebo groups (alpha 0.05, 2-sided significance). A two-sided *p*-value of less than 0.05 was considered statistically significant. All statistical analyses were performed using Stata 16.0 (StataCorp LP., College Station, TX, United States).

## Results

Of the 304 participants randomised to receive atorvastatin (*n* = 151) or placebo (*n* = 153), 248 (81.6%) participants completed the study (Supplementary Figure 1) (15). Participant characteristics at baseline are shown in Table 1. The mean age was 55.7 (SD 7.6) years, and 169 (55.6%) were women. There were no significant between-group differences for age, body mass index, severity of radiographic knee OA, muscle strength, vastus medialis CSA, or CK levels. The atorvastatin group had a higher proportion of females (*p* = 0.06), lower ALT (*p* = 0.04) and AST (*p* = 0.04) levels than the placebo group. Baseline characteristics of participants who completed the study and those who dropped out are presented in Supplementary Table 1. Participants who dropped out in the atorvastatin group were significantly younger than those who completed the study (*p* < 0.001). Participants who dropped out in the placebo group had higher ALT (*p* = 0.04) and AST (*p* = 0.02) levels than those who completed the study. Reasons for dropouts are presented in Supplementary Table 2.

**TABLE 1:** Baseline characteristics of study participants.

	Total population *N* = 304	Atorvastatin *N* = 151	Placebo *N* = 153	*P*
Age, years	55.7 (7.6)	55.7 (7.3)	55.8 (7.9)	0.89
Female, *n* (%)	169 (55.6)	92 (60.9)	77 (50.3)	0.06
Body mass index, kg/m^2^	29.4 (5.8)	29.4 (5.7)	29.5 (5.8)	0.85
Joint space narrowing^a^, *n* (%)				0.97
Grade 0	131 (44.3)	64 (43.8)	67 (44.7)	
Grade 1	102 (34.5)	52 (35.6)	50 (33.3)	
Grade 2	63 (21.3)	30 (20.6)	33 (22.0)	
Muscle strength^b^, kg	84.5 (49.7)	81.0 (46.3)	88.0 (52.9)	0.23
Vastus medialis cross-sectional area^c^, cm^2^	11.0 (3.4)	10.8 (3.2)	11.2 (3.6)	0.29
CK^d^, U/L	93.5 (70, 130)	91 (70, 126)	96 (70, 133)	0.37
ALT^e^, U/L	21 (16, 28)	19 (14, 26)	21 (16, 29.5)	0.04
AST^f^, U/L	20 (17, 24)	19.5 (16, 23)	21 (18, 25)	0.04

Data presented as mean (standard deviation), no (%), or median (interquartile range). ^a^*n* = 296; ^b^*n* = 290; ^c^*n* = 301; ^d^*n* = 298; ^e^*n* = 303; ^f^*n* = 302. ALT, alanine aminotransferase; AST, aspartate aminotransferase; CK, creatinine kinase.

### Muscle biochemistry

**FIGURE 1:**
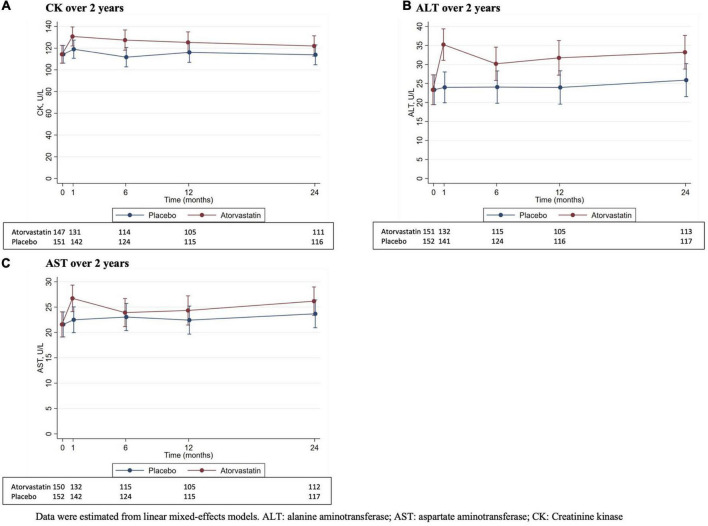
Muscle biochemistry biomarkers (mean and 95% confidence interval) at each time point over 2 years. Data were estimated from linear mixed-effects models. ALT, alanine aminotransferase; CK, creatinine kinase.

Figure 1 and Table 2 show the effect of atorvastatin on muscle biochemistry biomarkers and their changes over 2 years. There were no significant between-group differences in CK levels at all timespoints (Figure 1A and Table 2). The change in CK levels at 6 months from baseline was higher in the atorvastatin group compared with the placebo group (*p* = 0.04) with no significant between-group differences at other timespoints (Table 2). Although ALT levels were lower in the atorvastatin group compared with the placebo group at baseline, the atorvastatin group had higher ALT levels than the placebo group at 4 weeks (*p* = 0.004) and 6 months (*p* = 0.007). The between-group differences in ALT levels were not statistically significant at 12 and 24 months (Figure 1B and Table 2). The change in ALT levels from baseline was higher in the atorvastatin group compared with the placebo group at 4 weeks (*p* = 0.001), 12 (*p* = 0.03) and 24 (*p* = 0.03) months (Table 2). Despite higher AST levels in the placebo group than the atorvastatin group at baseline, there were no significant between-group differences in AST levels at all follow-up timespoints (Figure 1C and Table 2). The change in AST levels from baseline was higher in the atorvastatin group compared with the placebo group at 4 weeks (*p* = 0.04) with no significant between-group differences at other timespoints (Table 2).

**TABLE 2:** Muscle biochemistry biomarkers over 2 years.

	CK, U/L			ALT, U/L			AST, U/L		
	Atorvastatin	Placebo	*P*	Atorvastatin	Placebo	*P*	Atorvastatin	Placebo	*P*
**Baseline** Median (IQR)	91 (70, 126)	96 (70, 133)	0.37^a^	19 (14, 26)	21 (16, 29.5)	0.04^b^	19.5 (16, 23)	21 (18, 25)	0.04^c^
**4 weeks** Median (IQR)	107 (74, 157)	110 (75, 142)	0.76^d^	26 (19.5, 35)	21 (17, 29)	0.004^d^	22 (18.5, 26.5)	21 (17, 26)	0.14^e^
**6 months** Median (IQR)	109 (76, 149)	101.5 (72, 136)	0.37^f^	25 (19, 34)	22 (15, 30)	0.007^g^	21 (18, 26)	20 (18, 26)	0.45^g^
**12 months** Median (IQR)	103 (78, 140)	103 (72, 148)	0.93^h^	24 (19, 30)	21 (16, 30)	0.08^i^	22 (17, 26)	21 (17, 27)	0.99^h^
**24 months** Median (IQR)	103 (78, 139)	93.5 (62, 136)	0.17^j^	24 (19, 32)	21 (16, 29)	0.053^k^	22 (19, 26)	21 (17, 26)	0.34^l^
**Change from baseline to 4 weeks** Mean (95% CI)*	16.4 (6.3, 26.6)	4.7 (−5.1, 14.5)	0.10	11.9 (7.3, 16.4)	0.6 (−3.8, 5.1)	0.001	5.1 (2.2, 8.0)	0.9 (−1.9, 3.7)	0.04
**Change from baseline to 6 months** Mean (95% CI)*	13.1 (2.5, 23.7)	−2.6 (−12.8, 7.5)	0.04	6.8 (2.1, 11.6)	0.7 (−3.9, 5.3)	0.07	2.3 (−0.7, 5.4)	1.5 (−1.5, 4.4)	0.69
**Change from baseline to 12 months** Mean (95% CI)*	11.0 (0.1, 21.9)	1.8 (−8.7, 12.2)	0.23	8.4 (3.5, 13.3)	0.6 (−4.2, 5.4)	0.03	2.8 (−0.4, 5.9)	0.9 (−2.2, 3.9)	0.40
**Change from baseline to 24 months** Mean (95% CI)*	7.7 (−2.9, 18.4)	−0.5 (−10.9, 9.9)	0.28	9.9 (5.1, 14.7)	2.5 (−2.2, 7.3)	0.03	4.6 (1.5, 7.6)	2.1 (−0.9, 5.1)	0.26
									

^a^*n* = 298; ^b^*n* = 303; ^c^*n* = 302; ^d^*n* = 273; ^e^*n* = 274; ^f^*n* = 238; ^g^*n* = 239; ^h^*n* = 220; ^I^*n* = 221; ^j^*n* = 227; ^k^*n* = 230; ^l^*n* = 229. ALT, alanine aminotransferase; AST, aspartate aminotransferase; CK, creatinine kinase; IQR, interquartile range; CI, confidence interval.

*Linear mixed-effects models adjusted for sex and respective baseline value.

### Muscle strength and size

The effect of atorvastatin on muscle strength, size, and their changes over 2 years are shown in Table 3. There was a significant increase in muscle strength in the atorvastatin group over 12 and 24 months and in the placebo group over 24 months. However, the change in muscle strength was not significantly different between the two groups. Although no significant change in vastus medialis CSA was observed over 24 months in either group, there was significant between-group difference in the change of vastus medialis CSA (0.11 vs. −0.23, *p* = 0.02).

**TABLE 3:** Muscle strength and size over 2 years.

	**Muscle strength, kg**			**Muscle CSA, cm^2^**		
	**Atorvastatin**	**Placebo**	***P***	**Atorvastatin**	**Placebo**	***P***
**Baseline** Mean (SD)	81.0 (46.3)	88.0 (52.9)	0.23^a^	10.8 (3.2)	11.2 (3.6)	0.29^d^
**12 months** Mean (SD)	87.6 (38.6)	91.1 (51.1)	0.55^b^	–	–	–
**24 months** Mean (SD)	90.5 (40.0)	91.9 (48.7)	0.81^c^	10.8 (3.4)	10.8 (3.3)	0.95^e^
**Change from baseline to 12 months** Mean (95% CI)*	6.1 (1.4, 10.8)	2.1 (−2.4, 6.7)	0.23	–	–	–
**Change from baseline to 24 months** Mean (95% CI)*	8.2 (3.5, 12.9)	5.9 (1.3, 10.4)	0.49	0.11 (−0.10, 0.31)	−0.23 (−0.43, −0.03)	0.02
						

^a^*n* = 290; ^b^*n* = 238; ^c^*n* = 237; ^d^*n* = 301; ^e^*n* = 244. CSA, cross-sectional area; SD, standard deviation; CI, confidence interval.

*Linear mixed-effects models adjusted for sex and respective baseline value.

### Incidence of myalgia

Table 4 shows the incidence of myalgia at different timespoints throughout the study. The incidence of myalgia was slightly higher in the atorvastatin group than the placebo group over 2 years (8/151 vs. 2/153, *p* = 0.06). Most of the myalgia occurred within the first 6 months after drug commencement (7/151 vs. 1/153, *p* = 0.04).

**TABLE 4:** Incidence of myalgia.

	Atorvastatin, *n* = 151	Placebo, *n* = 153	*P*
Myalgia within 4 weeks, *n* (%)	2 (1.3)	0	0.25
Myalgia 4 weeks–6 months, *n* (%)	5 (3.3)	1 (0.7)	0.12
Myalgia 6–12 months, *n* (%)	1 (0.6)	1 (0.7)	1.00
Total, *n* (%)	8 (5.3)	2 (1.3)	0.06

### Relationship between creatinine kinase levels and myalgia

Characteristics of participants who developed myalgia are presented in Supplementary Table 3. There was no relationship between the incidence of myalgia and CK levels. Of the 10 participants (8 in atorvastatin group and 2 in placebo group), the majority had normal levels of CK, AST, and ALT. Only 2 participants in the atorvastatin group had mildly elevated levels of CK, <1.5 times the upper limit of normal. Participants described their symptoms as muscle cramps, aches, unilateral calf pain, or severe muscle pain with weakness. CK levels were within normal limit in the participant who reported severe muscle pain with weakness. Two participants (one in each group) had underlying thyroid disease.

## Discussion

We showed in this *post-hoc* analysis of a randomised placebo-controlled trial, that in participants with symptomatic knee OA, atorvastatin 40 mg daily had no adverse effect on muscle biochemistry, strength or size, despite a slightly higher incidence of myalgia over 2 years that usually occurred within 6 months of drug commencement. As such, given the OA population has twice the risk of cardiovascular death than the general population, clinicians should not withhold the substantial benefit of statins in OA populations, especially when dealing with mild statin-associated muscle symptoms.

Muscle biochemistry biomarkers, including CK and AST, muscle strength and size were not affected by high-intensity atorvastatin dose (40 mg daily) in people with symptomatic knee OA. No participants developed myopathy or myositis. Our study found no significant between-group differences in CK levels at all timespoints. This is in contrast to the Effects of Statins on Skeletal Muscle Function and Performance (STOMP) trial that showed a small (∼20 U/L) but significant (*p* < 0.01) increase in CK levels at 6 months with atorvastatin 80 mg among healthy, statin-naïve participants (21). Among our eight participants assigned to atorvastatin who developed myalgia, there was no significant increase in CK levels. There is evidence for a dose-dependent effect of statins on statin-induced muscle symptoms, such that high dose statins produce a 10-fold higher rate of myopathy development than a low dose statin (8, 22). This may explain the differences in CK findings between our study and the STOMP trial. In our study despite a statistically significant higher ALT levels in the atorvastatin group at 4 weeks and 6 months compared with the placebo group which diminished after 6 months, these changes were not clinically significant. Of those who had abnormal ALT levels at 4 weeks (24/151 in atorvastatin group vs. 15/153 in placebo group), only 2 participants in the atorvastatin group had ALT levels of 3 times the upper limit of normal. These ALT abnormalities were transient and were all resolved by 6 months, with no participants having ALT levels of 3 times the upper limit of normal at 6 months after drug commencement. Although ALT is usually present in the liver at a much higher concentration, it can also be found in skeletal muscles (23). Its levels tend to stabilise despite continuation of treatment, as seen in our study, and most likely represent adaptation of the liver to the lower serum cholesterol, rather than direct hepatotoxicity (24, 25).

The evidence regarding the effect of statins on muscle strength, function and performance is conflicting (14). Our study showed high-intensity atorvastatin dose had no adverse effect on muscle strength and size. In fact, we found increased muscle strength at 12 and 24 months in the atorvastatin group, while in the placebo group increased muscle strength was observed at 24 months but not 12 months. In contrast, a previous study showed that self-reported statin use in older adults (mean age 62 years) was associated with significantly reduced leg strength, and that those remaining on statin use at baseline and follow-up demonstrated significantly lower leg strength than those who ceased statin therapy (14). The participants in the previous study were older and had more comorbidities (63.9% of statin users had CVD and 12.9% had diabetes) than in our study and the dosage of statin was unknown. It may be that other factors such as age-related neuromuscular decline, may explain the lower leg strength in statin users. In support of our findings, the STOMP trial which examined healthy people without OA, also showed no detrimental effect on muscle strength or exercise performance with high dose 80 mg atorvastatin (21).

Consistent with evidence from previous clinical trials (10, 21), we found that high-intensity atorvastatin dose was associated with a trend to a higher incidence of myalgia over 2 years, usually occurring within 6 months of drug commencement. Within the statin drug class, atorvastatin has been associated with higher incidence of myalgia compared to placebo (8, 21). However, most of the concerns arise from significantly higher incidence of myalgia noted in observational studies rather than that reported in randomised controlled trials (10, 25). Additionally, most clinical trials excluded participants with chronic pain, such as those with symptomatic OA. Encouragingly, we showed the incidence of myalgia from high-intensity dose of atorvastatin in patients with symptomatic knee OA was 5.3% (8/151), which was similar to other non-OA clinical trials (8, 10). In real-life clinical practice, statins are often discontinued because of their “perceived” side effects (7), in particular related to skeletal muscle. Although we did not show any significant relationship between CK levels and myalgia in our analysis, of those who developed myalgia, 50% (3 of 8 in the atorvastatin group and 2 of 2 in the placebo group) discontinued therapy. The magnitude of this potential nocebo effect was elegantly evaluated in the N-of-1 trial that showed 90% of the symptom burden caused by statin was also elicited by placebo and 50% of them were able to successfully restart statins (26).

People with OA are twice more likely to die from CVD than the age-matched general population (2, 27), owing to the high prevalence of shared traditional CVD risk factors, including dyslipidaemia (1). The benefit of reduction in low-density lipoprotein cholesterol on CVD events is well documented, such that for every 1 mmol/L reduction, there is a significant 22% reduction in the risk of major vascular and coronary events, regardless of the baseline level (5, 28). Given the increased risk of CVD death in those with OA, there is a need to target CVD risk factors in those with OA. This study provides reassuring data of the safety of high-intensity atorvastatin on skeletal muscles in those with symptomatic knee OA, despite a slightly increased incidence of myalgia symptoms. In our group of participants with low-to-medium CVD risk, we found no adverse effects of atorvastatin on muscle properties including muscle biochemistry, strength, or size, particularly when we focused on lower limb muscles that are significantly affected in those with symptomatic knee OA. Hence, the substantial benefit of statin in people with OA should not be held back.

This study has limitations. As it was a *post-hoc* analysis of a randomised controlled trial, the inherent issue of statin-induced myalgia incidence discrepancy between observational studies and randomised controlled trials remains as participants in this study were highly selected. However, in our study, we targetted a population with symptomatic knee OA, hence addressing a significant clinical gap on statin safety in a group with high CVD risk. Additionally, we showed no differences in baseline muscle properties between those who dropped out and those who completed the study (Supplementary Table 1). Our study population was limited to those without a valid indication for statin use, as it would be unethical to withhold statin with a clinical indication, for example those with estimated high cardiovascular risk, currently on lipid-lowering therapy, or with fasting total cholesterol level >7.5 mmol/L (who often have familial hypercholesterolaemia) were excluded. It is likely that those with knee OA who were excluded from this study, are the population at greatest need for statins. At the same time, this population also generally has more comorbidities requiring other concomitant drugs, and thus is at an increased risk of statin toxicity (8). Therefore, our study may have underestimated the potential muscle-related adverse effect of statins in people with OA. However, we showed that high-intensity atorvastatin dose had no adverse effects on skeletal muscle in people with symptomatic knee OA with low-to-medium CVD risk. One of the strengths of this study is that we recruited participants from the community. Since knee OA is common, with 48% of community-based adults (mean age 63 years, range 50–79 years) having knee pain (29), this increases the generalisability of our findings to the broad population with symptomatic knee OA. Apart from self-reported myalgia, all other muscle measures were objectively assessed, including using laboratory tests for muscle biochemistry, MRI for muscle size, and dynamometer for muscle strength.

In conclusion, we showed that high-intensity atorvastatin at a dose of 40 mg once daily had no adverse effect on muscle biochemistry, strength and size among participants with symptomatic knee OA, apart from a slightly higher incidence of myalgia over 2 years, usually occurring within 6 months of drug commencement. Given the OA population is known to be at higher risk of cardiovascular morbidities and mortality than the general population, with the findings of this study, the substantial benefit of statins in OA populations should not be withheld (3, 5).

## Data availability statement

The datasets presented in this article are not readily available because the data generated from this study will not be deposited in a public repository due to privacy and consent restrictions. De-identified data can be made available from the corresponding author on reasonable request, subject to a data sharing agreement. Requests to access the datasets should be directed to YW, yuanyuan.wang@monash.edu.

## Ethics statement

The studies involving human participants were reviewed and approved by the Alfred Hospital Ethics Committee, Monash University Human Research Ethics Committee, Tasmania Health and Medical Human Research Ethics Committee, and The Queen Elizabeth Hospital Human Research Ethics Committee. The patients/participants provided their written informed consent to participate in this study.

## Author contributions

YL: analysis and interpretation of the data, drafting of the manuscript, and final approval of the manuscript. FC and YW: conception and design, analysis and interpretation of the data, critical revision of the manuscript for important intellectual content, and final approval of the manuscript. AW, GJ, CH, AT, and CD: interpretation of the data, critical revision of the manuscript for important intellectual content, and final approval of the manuscript. AF: analysis and interpretation of the data, critical revision of the manuscript for important intellectual content, and final approval of the manuscript. SB: acquisition of data, interpretation of the data, critical revision of the manuscript for important intellectual content, and final approval of the manuscript. LT: conception, critical revision of the manuscript for important intellectual content, and final approval of the manuscript. All authors contributed to the article and approved the submitted version.
