# Supplementary material for: Effect of atorvastatin on skeletal muscles of patients with knee osteoarthritis: Post-hoc analysis of a randomised controlled trial
Source: Front Med (Lausanne). 2022 Aug 25;9:939800. doi: 10.3389/fmed.2022.939800 (PMC9452814; doi:10.3389/fmed.2022.939800)
Supplement: Supplementary file 1 [file Data_Sheet_1.PDF]

Supplemental Figure 1: Study flow chart

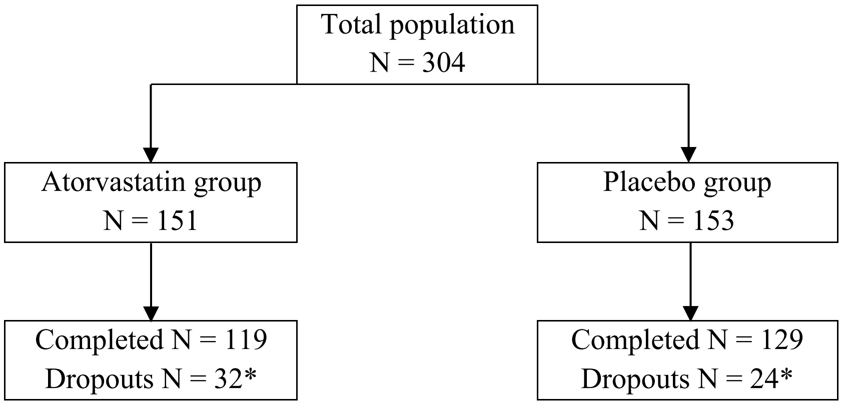

* Reasons for dropouts are presented in Supplemental Table 2

Supplemental Table 1: Baseline characteristics of study participants

	Atorvastatin (n=151)			Placebo (n=153)		
	Completed n=119	Dropouts n=32	p	Completed n=129	Dropouts n=24	p
Age, years	56.8 (7.2)	51.4 (6.2)	<0.001	56.2 (7.9)	53.5 (7.5)	0.13
Female, n (%)	72 (60.5)	20 (62.5)	0.84	66 (51.2)	11 (45.8)	0.63
Body mass index, kg/m^2	29.6 (5.5)	28.5 (6.2)	0.32	29.4 (5.9)	30.0 (5.3)	0.68
Joint space narrowing*, n (%)			0.19			0.76
Grade 0	47 (40.9)	17 (54.8)		55 (43.7)	12 (50.0)	
Grade 1	41 (35.6)	11 (35.5)		42 (33.3)	8 (33.3)	
Grade 2	27(23.5)	3 (9.7)		29 (23.0)	4 (16.7)	
Muscle strength, kg	81.4 (43.3)[a]	79.9 (56.2)	0.87	85.3 (53.5)[b]	102.0 (48.7)	0.16
Vastus medialis cross-sectional area, cm^2	10.6 (3.3)[c]	11.2 (2.9)	0.36	11.0 (3.5)[d]	12.1 (4.0)	0.18
CK, U/L, median (IQR)	91 (70, 123.5)[e]	92 (69, 156)	0.47	95 (68, 133)[d]	119.5 (80.5, 141.5)	0.17
ALT, U/L, median (IQR)	19 (14,26)	18.5 (14.25, 30.5)	0.87	21 (16, 27)[f]	25.5 (20, 38.5)	0.04
AST, U/L, median (IQR)	19 (16,23)[c]	20.5 (18, 24.75)	0.17	20.5 (17, 24.75)[f]	23.5 (19.25, 28.75)	0.02

Data presented as mean (standard deviation) or no (%)

*n=296 (atorvastatin group n=146; placebo group n=150); [a]n=113; [b]n=121; [c]n=118; [d]n=127; [e]n=116; [f]n=128

ALT: alanine aminotransferase, AST: aspartate aminotransferase, CK: creatinine kinase, IQR: interquartile range

Supplemental Table 2: Reasons for dropouts (early withdrawal)

Withdrawal reason	No	Comments
Had a knee replacement or due to have a knee replacement	5	
Started statins or other lipid lowering medication	4	
Dropped out due to other event/reason	22	Due to medical reasons, n=11<br>Side effects, n=6<br>Due to social reason, n=1<br>Not specified, n=3<br>Unable to have magnetic resonance imaging, n=1
Participant has moved away from study site	2	
No longer wishes to continue in the study for personal or other reasons	15	
Cannot be contacted	8	
Total	56	

Supplemental Table 3: Characteristics of participants who developed myalgia

Myalgia onset, days*	Atorvastatin / Placebo	Symptom description	CK, U/L**	ALT, U/L	AST, U/L	Discontinue (Y/N)	Age	Vigorous physical activity (Y/N)	Comments
18	Atorvastatin	Calf pains	152	29	15	N	59.7	Y	
732		Right thigh calf cramp and pain	284	37	20				
26	Atorvastatin	Muscle aches, felt unwell	122	35	21	Y	48.9	Y	
84	Atorvastatin	Muscle pain (aches) without weakness, ongoing cramps	89	17	18	Y	62.6	N	
91	Placebo	Muscle cramps	NA	NA	NA	Y	67.1	Y	Withdrawal from study at 4 weeks
107	Atorvastatin	Fairly severe muscle pain and weakness in first few months. Resolved without intervention.	66	19	17	N	66.5	N	On thyroxine for hypothyroidism
109	Atorvastatin	Muscle aches	54	NA	NA	Y	57.0	Y	Withdrawal from study at 4 weeks
134	Atorvastatin	Right leg muscle soreness	50	12	11	N	65.4	Y	
144	Atorvastatin	Muscle soreness	132	17	21	N	66.6	Y	
182	Placebo	Left calf pain	139	NA	NA	Y	51.3	N	On thyroxine post total thyroidectomy. Withdrawal from study at 6 months, unrelated to calf pain (due to some other medical issues)
708	Atorvastatin	Significant muscle spasm	247	33	27	N	58.5	N	Had elevated CK (1.4x ULN) 3 months after commencement of drug, however clinically asymptomatic and went to gym for intensive exercise prior to blood tests. After developing muscle spasm, CK remained elevated for approximately 11 months at 1.4x ULN.

* Days post randomisation
** CK results within 3 months of myalgia onset.
ALT: alanine aminotransferase; AST: aspartate aminotransferase; CK: creatinine kinase; N: no; NA: not available; ULN: upper limit normal; Y: yes
